# Complete Genome Sequence of a Vampire Bat Rabies Virus from French Guiana

**DOI:** 10.1128/genomeA.00188-16

**Published:** 2016-04-07

**Authors:** Anne Lavergne, Edith Darcissac, Hervé Bourhy, Sourakhata Tirera, Benoît de Thoisy, Vincent Lacoste

**Affiliations:** a Laboratoire des Interactions Virus-Hôtes, Institut Pasteur de la Guyane, Cayenne, French Guiana; b Institut Pasteur, Lyssavirus Dynamics and Host Adaptation Unit, National Reference Centre for Rabies, WHO Collaborating Centre for Reference and Research on Rabies, Paris, France

## Abstract

A rabies virus was detected in a common vampire bat (*Desmodus rotundus*) in French Guiana. Its genomic sequence was obtained and found to be closely related to other hematophagous bat-related viruses that widely circulate in the northern Amazon region. This virus is named AT6.

## GENOME ANNOUNCEMENT

Rabies is a viral disease distributed all over the world, causing more than 55,000 deaths per year, principally in Africa and Asia (1). Most of the time, it is transmitted via rabid domestic animals, but the virus is also maintained and transmitted through sylvatic reservoirs, of which bats are a member. Vampire bats are now considered the main vector of the disease in South America (2, 3). In French Guiana, on the northern part of South America, 14 rabies cases were recorded in cattle and domestic carnivores between 1984 and 2011 (4). In 2008, a human rabies case was recorded (5). All cases were related to vampire bat-associated rabies viruses (RABV). RABV belongs to the *Rhabdoviridae* family, genus *Lyssavirus*. Its genome is a single-stranded negative-sense RNA of approximately 12 kb that encodes five structural proteins. A previous work on the circulation of rabies virus in wild bats in French Guiana allowed us to identify, among nearly 1,000 bats belonging to 30 species, one common vampire bat, *Desmodus rotundus*, which was positive for RABV (6).

We report here the full-genome sequence of the rabies virus isolate AT6. To generate the complete genome sequence, total RNA was extracted from the heart using the NucliSENS easyMAG bio-robot (bioMérieux). cDNA was prepared with the SuperScript III reverse transcriptase (Invitrogen). Sequences were generated by reverse transcription PCR (RT-PCR) using different combinations of primers. Overlapping amplicons were generated, cloned, and then sequenced by Beckman Coulter Genomics (Takeley, United Kingdom). One contig sequence was assembled using the MEGA5 software (7).

The complete genome of the strain is 11,922 nucleotides (nt) in length. Its genetic organization is consistent with that of previously sequenced rabies virus genomes, with five open reading frames unidirectionally transcribed and separated by intergenic regions of various sizes (8). The coding sequences are 1,353 nt for the nucleoprotein-encoding gene, 894 nt for the phosphoprotein, 609 nt for the matrix protein, 1,575 nt for the glycoprotein, and 6,387 nt for the RNA-dependent RNA polymerase, with the RNA-dependent RNA polymerase having a double start codon characteristic of RABV strains isolated from bats in the Americas (9).

We conducted a phylogenetic analysis based on the complete nucleotide sequence using a Bayesian approach with previously published rabies virus sequences. It showed that AT6 is closely related to a strain (accession no. KM594041) isolated from a *D. rotundus* bat in Brazil in 2013 with 98.79% nucleotide identity. These two sequences belong to a clade composed of sequences of desmodine origin from Brazil and French Guiana identified during the last two decades. All these strains belong to a large group of hematophagous bat-related viruses, named lineage II, which widely circulate in the northern Amazon region (10). Further analysis of RABV genomes detected in the northern Amazon region should enable an understanding of the circulation and transmission of RABV lineages in the bat populations.

### Nucleotide sequence accession number.

The complete genome sequence of RABV AT6 is available in the GenBank database under accession no. KU523255.
